# Crosstalk Between DNA Methylation and Gene Mutations in Colorectal Cancer

**DOI:** 10.3389/fonc.2021.697409

**Published:** 2021-07-01

**Authors:** Maria Dobre, Alessandro Salvi, Iulia Andreea Pelisenco, Florina Vasilescu, Giuseppina De Petro, Vlad Herlea, Elena Milanesi

**Affiliations:** ^1^ Laboratory of Histopathology and Immunohistochemistry, Victor Babes National Institute of Pathology, Bucharest, Romania; ^2^ Division of Biology and Genetics, Department of Molecular and Translational Medicine, University of Brescia, Brescia, Italy; ^3^ Faculty of Biology, University of Bucharest, Bucharest, Romania; ^4^ Department of Pathology, Fundeni Clinical Institute, Bucharest, Romania; ^5^ Laboratory of Radiobiology, Victor Babes National Institute of Pathology, Bucharest, Romania

**Keywords:** colorectal cancer, DNA methylation, *KRAS*, *BRAF*, mutations

## Abstract

Colorectal cancer (CRC) is often characterized by mutations and aberrant DNA methylation within the promoters of tumor suppressor genes and proto-oncogenes. The most frequent somatic mutations occur within *KRAS* and *BRAF* genes. Mutations of the *KRAS* gene have been detected in approximately 40% of patients, while mutations in *BRAF* have been detected less frequently at a rate of 10%. In this study, the DNA methylation levels of 22 candidate genes were evaluated in three types of tissue: mucosal tumoral tissue from 18 CRC patients, normal adjacent tissues from 10 CRC patients who underwent surgical resection, and tissue from a control group of six individuals with normal colonoscopies. A differential methylation profile of nine genes (*RUNX3*, *SFRP1*, *WIF1*, *PCDH10*, *DKK2*, *DKK3*, *TMEFF2*, *OPCML*, and *SFRP2*) presenting high methylation levels in tumoral compared to normal tissues was identified. *KRAS* mutations (codons 12 or 13) were detected in eight CRC cases, and *BRAF* mutations (codon 600) in four cases. One of the CRC patients presented concomitant mutations in *KRAS* codon 12 and *BRAF*, whereas seven patients did not present these mutations (WT). When comparing the methylation profile according to mutation status, we found that six genes (*SFRP2*, *DKK2*, *PCDH10*, *TMEFF2*, *SFRP1*, *HS3ST2*) showed a methylation level higher in *BRAF* positive cases than *BRAF* negative cases. The molecular sub-classification of CRC according to mutations and epigenetic modifications may help to identify epigenetic biomarkers useful in designing personalized strategies to improve patient outcomes.

## Introduction

Colorectal cancer (CRC) is the third most common type of cancer and, even with advances in CRC screening and therapeutic strategies, it still remains the second deadliest malignancy for both sexes. CRC incidence has continued to increase in countries with medium to high human development indexes and in younger populations (1). This type of cancer is a highly heterogeneous disease that can be subtyped according to anatomical location or pathological and molecular signatures. The development of CRC is influenced by both environmental risk factors (such as obesity, a sedentary lifestyle, an unhealthy diet, alcohol consumption, and smoking) (2) and genetic risk factors, with less than 10% of patients presenting inherited mutations that increase the risk of CRC onset (3) and 25% of patients with “familial” CRC (4). About 70% of CRC cases are sporadic; these cases are most common in patients over the age of 50 and seem to depend mainly on dietary and environmental factors.

In general, the development of sporadic CRCs involves the “normal-adenoma-dysplasia-carcinoma” sequence as described by Vogelstein and Fearon (5). This sequence includes genetic and epigenetic changes in the colonic epithelium that transform normal glandular epithelium into invasive adenocarcinomas. The progression from adenoma to carcinoma is a multistep process that involves three molecular pathways: the Chromosomal Instability (CIN) pathway, the Microsatellite Instability (MSI) pathway, and the CpG Island Methylator Phenotype (CIMP) pathway. CIN is characterized by loss or gain of chromosomal segments, chromosomal translocations, or gene amplifications, which result in gene copy number variations. In addition, mutations in specific oncogenes, including *KRAS* and *BRAF*, and in tumor suppressor genes, such as *APC* and *TP53*, can be detected (6). MSI occurs in 15–20% of sporadic CRC cases and comprises recurrent alterations in the microsatellite zone without structural and numerical changes in the genome. CIMP, reported in 20–30% of sporadic CRCs (7), is characterized by hyper-methylation of CpG islands located in promoters that regulate the activity of several tumor suppressor genes and other CRC related genes. It is well known that specific mutations can modify DNA methylation (8) and that DNA methylation changes can cause an increase in mutation rate (9). In CRC, 10–40% lower levels of absolute methylation than normal colon tissue within the whole genome have been determined (10). However, CpG islands in the promoters of CRC related genes show a hyper-methylated profile, resulting in repression of transcription of tumor suppressor genes (11). Therefore, the first aim of this study was to identify methylation differences in the promoters of 22 candidate genes in CRC tissues compared with control tissues (from non-CRC individuals) and with paired normal adjacent tissues. The second aim was to examine the relationship between promoter DNA methylation and the presence of mutations in *KRAS* and *BRAF* genes.

## Materials and Methods

### Sample Collection

The DNA for the study was obtained from 18 CRC sporadic tumor tissues and 10 corresponding paired normal adjacent tissues (NAT) from CRC patients who underwent surgical resection. The NATs were collected approximatively 10 cm away from the tumors. For nine patients out of 10, the NAT was collected from the same anatomic segment. For one patient with tumor localization in the recto-sigmoid junction, the NAT was collected from the sigmoid. For five of the 18 CRC patients, blood samples were collected in EDTA tubes. The tissue specimens and the blood samples were collected at Fundeni Clinical Institute in Bucharest (Romania) and were stored at −80°C until DNA isolation. Six specimens from normal colonic mucosa as well as five blood samples from independent controls, from previous preclinical studies were available from the biobank of the Victor Babes Institute (**Table 1**). The present study was approved by the local ethics committee (registration number 291, 8 March 2016), carried out in accordance with the Declaration of Helsinki, and the individuals gave their written informed consent. Genomic DNA was isolated using the QIAmp DNA Mini Kit (Qiagen) according to the manufacturer’s instruction and quantified using a NanoDrop 2000 spectrophotometer (Thermo Scientific). All samples were examined by a pathologist, and clinical information of the patient’s cohort are reported in **Table 2**.

**Table 1:** Socio-demographic data of the individuals involved in the study.

Group	Age ± SD	Sex (% Female)	p-value Age	p-value, Sex
**T (N = 18)**	65.72 ± 11.31	55.55	T *vs* C, p = 0.381	T *vs* C, p = 0.633, χ^2^ = 0.229
**NAT (N = 10)**	62.40 ± 11.16	72.72	T *vs* PT, p = 0.461	T *vs* PT, p = 0.453, χ^2^ = 0.562
**C (N = 6)**	60.66 ± 14.06	66.66	PT *vs* C, p = 0.789	PT *vs* C, p = 0.793, χ^2^ = 0.069

**Table 2:** Available clinical data and mutation status of CRC patients involved in the study.

Mutational status (KRAS/BRAF)	Number	Mutation type	Location	Hepatic metastasis
**WT**	7	//	Colon (n = 3)	1
			Rect/RSJ (n = 3)	
			Sigmoid (n = 1)	
**KRAS12+**	4	KRAS12_G12D (n = 2)	Colon (n = 3)	1^*^
		KRAS12_G12V^*^ (n = 2)	Sigmoid (n = 1)	
**KRAS13+**	3	KRAS13_G13D (n = 3)	Colon (n = 1)	0
			Rect/RSJ (n = 1)	
			Sigmoid (n = 1)	
**BRAF+**	3	BRAF_V600E (n = 3)	Colon (n = 2)	1
			Rect/RSJ (n = 1)	
**KRAS12+BRAF+**	1	KRAS12_G12D+ BRAF_V600E (n = 1)	Sigmoid (n = 1)	0

*The patient with hepatic metastasis presented KRAS12_G12V mutation.

### Detection of *KRAS* and *BRAF* Mutations Detection and DNA Methylation Analysis

*KRAS* gene mutations (in codons 12, 13, 61) were detected by pyrosequencing as previously described (12). Mutations in codons 600 and 601 of the *BRAF* gene were assessed using the BRAF 600/601 StripAssay (ViennaLab Diagnostic GmbH, Vienna, Austria) by PCR followed by reverse hybridization according to the manufacturer’s protocol. The DNA methylation levels of the promoters of the 22 genes involved in colorectal cancer (*APC*, *CDH1*, *CDKN2A*, *DKK2*, *DKK3*, *HIC1*, *HNF1B*, *HS3ST2*, *MGMT*, *MLH1*, *OPCML*, *PCDH10*, *RASSF1*, *RUNX3*, *SFRP1*, *SFRP2*, *SFRP5*, *SPARC*, *TMEFF2*, *UCHL1*, *WIF1*, and *WT1*) were analyzed using the EpiTect Methyl II PCR Array (Qiagen, Hilden, Germany) according to manufacturer’s protocol. This array system is based on the detection of the input DNA that remains after digestion with a methylation-sensitive and/or methylation-dependent restriction enzyme using the EpiTect Methyl II DNA Restriction kit (Qiagen, Hilden, Germany). The relative amount of un-methylated (UM) and methylated (M) DNA was quantified by qPCR using the comparative cycle threshold method.

### Statistical Analysis

The statistical analysis was conducted using the Statistical Package for Social Science (SPSS version 17.0). Categorical variables were tested using the chi-square test and continuous variables were tested using a t-test or Mann-Whitney U test. The Mann-Whitney U test was used to identify differences in promoter methylation levels between CRC patients (T) and controls (C), normal adjacent tissue (NAT) and controls (C), as well as to compare samples from patients with different mutation status for *KRAS* and *BRAF*. Receiver operating characteristic (ROC) curves were created and the area under the curve (AUC) was calculated to determine the role of methylation in differentiating T from C. Logistic regression was used to build a diagnostic model that could explore whether varying combinations of differentially methylated genes could better differentiate T from C. A gene promoter was considered to be methylated if the methylation level was over 20% according to the instructions of the manufacturer. A further analysis of the 10 tumoral samples with their matched NAT samples was performed using the Wilcoxon Signed Rank test. All reported significant p values were two-sided, with *p* < 0.05.

## Results

In this study, colonic mucosa sample from 18 patients with CRC and six controls were investigated to evaluate the levels of DNA methylation of the promoters of 22 target genes. The tumors were located in the ascending and transverse colon (n = 9), in the sigmoid (n = 4), and in the rectum or the rectosigmoid junction (RSJ) (n = 5). At the moment of sample collection, three cases presented hepatic metastasis. Seven out of the 18 analyzed tumoral tissues presented mutations in the *KRAS* gene: four occurred in codon 12 and three in codon 13. Three patients had mutations in the *BRAF* gene, and one patient showed a double mutation, i.e., mutations in both *BRAF* and *KRAS* genes. *KRAS* and *BRAF* mutations were not found in the 10 normal adjacent tissues or in the seven tumoral tissues (WT) (**Table 2**).

**Figure 1:**
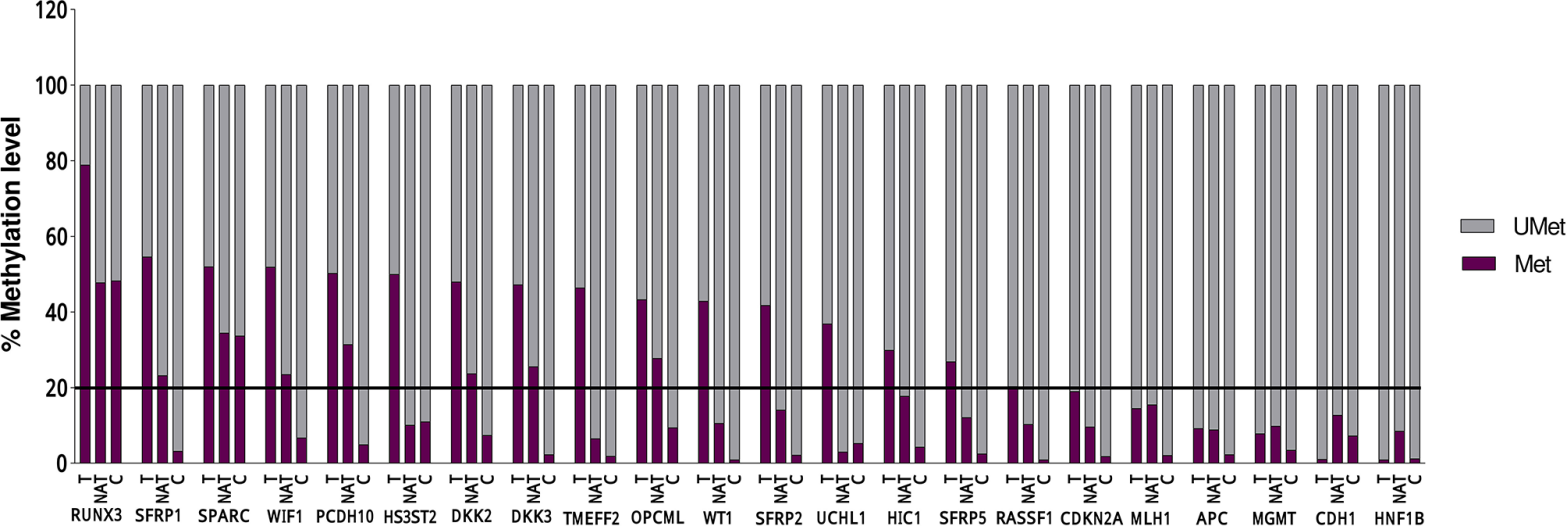
DNA methylation levels in 18 Tumoral (T), 10 Normal Adjacent Tissue l (NAT), and 6 Control (C) of each of the 22 evaluated genes. The line represent the threshold of 20% of CpG dinucleotides modified by a methyl group, above which the promoter is defined as methylated.

Statistical analysis of the DNA methylation levels of the promoters was performed for 16 genes out of 22, because in six genes, the detected DNA methylation level in the tumoral tissue was below 20% (**Figure 1** and **Table 3**).

**Table 3:** Average of the methylation percentage (± SEM) in tumoral and control tissues along with the statistical results between the two groups and the ROC curve analysis.

Gene	TUMORAL N = 18		CONTROL N = 6		*% Methylation T vs C*	*ROC*			
	Mean	SEM	Mean	SEM	*p-value (Mann-Whitney U test)*	AUC	Cut-off%	Sensitivity%/ Specificity%	*p-value*
**RUNX3**	78.87	4.05	48.31	4.83	***<0.001***	**0.917**	*55.57*	*94.4–83.3*	***0.003***
**SFRP1**	54.61	7.44	3.18	0.98	***<0.001***	**0.944**	*10.51*	*94.4–100*	***0.001***
**SPARC**	52.01	6.14	33.66	4.78	ns	* *	* *	* *	*** ***
**WIF1**	51.95	7.13	6.66	1.69	***0.003***	**0.889**	*15.69*	*88.9–100*	***0.005***
**PCDH10**	50.31	7.07	4.95	1.01	***0.001***	**0.935**	*12.03*	*88.9–100*	***0.002***
**HS3ST2**	50.03	8.39	11.04	8.7	ns	** **	* *	* *	*** ***
**DKK2**	47.96	6.68	7.38	1.58	***<0.001***	**0.981**	*13.81*	*94.4–100*	***0.001***
**DKK3**	47.22	8.65	2.35	0.55	***0.027***	**0.806**	*3.92*	*77.8–83.3*	***0.028***
**TMEFF2**	46.4	7.89	1.87	0.4	***0.002***	**0.907**	*2.99*	*88.9–83.3*	***0.003***
**OPCML**	43.28	5.37	9.38	2.69	***0.003***	**0.889**	*15.20*	*83.3–83.3*	***0.005***
**WT1**	42.9	9.09	0.9	0.23	*ns*		* *	* *	*** ***
**SFRP2**	41.84	7.51	2.18	0.66	***0.015***	**0.833**	*10.84*	*83.3–100*	***0.016***
**UCHL1**	36.95	7.6	5.28	2.65	ns	* *	* *	* *	*** ***
**HIC1**	29.87	8.12	4.34	0.58	ns	* *	* *	* *	*** ***
**SFRP5**	26.87	7.57	2.53	0.93	*ns*	* *	* *	* *	*** ***
**RASSF1**	20.28	6.6	0.87	0.22	ns	* *	* *	* *	*** ***
**CDKN2A**	19.03	7.09	1.84	0.38	Methylation <20%				
**MLH1**	14.48	6.88	2.05	0.95					
**APC**	9.2	3.36	2.28	0.58					
**MGMT**	7.81	3.38	3.5	1.54					
**CDH1**	1.08	0.1	7.35	2.38					
**HNF1B**	0.9	0.11	1.23	0.43					

The bold values refer to significant results. NS, not significant.

**Figure 2:**
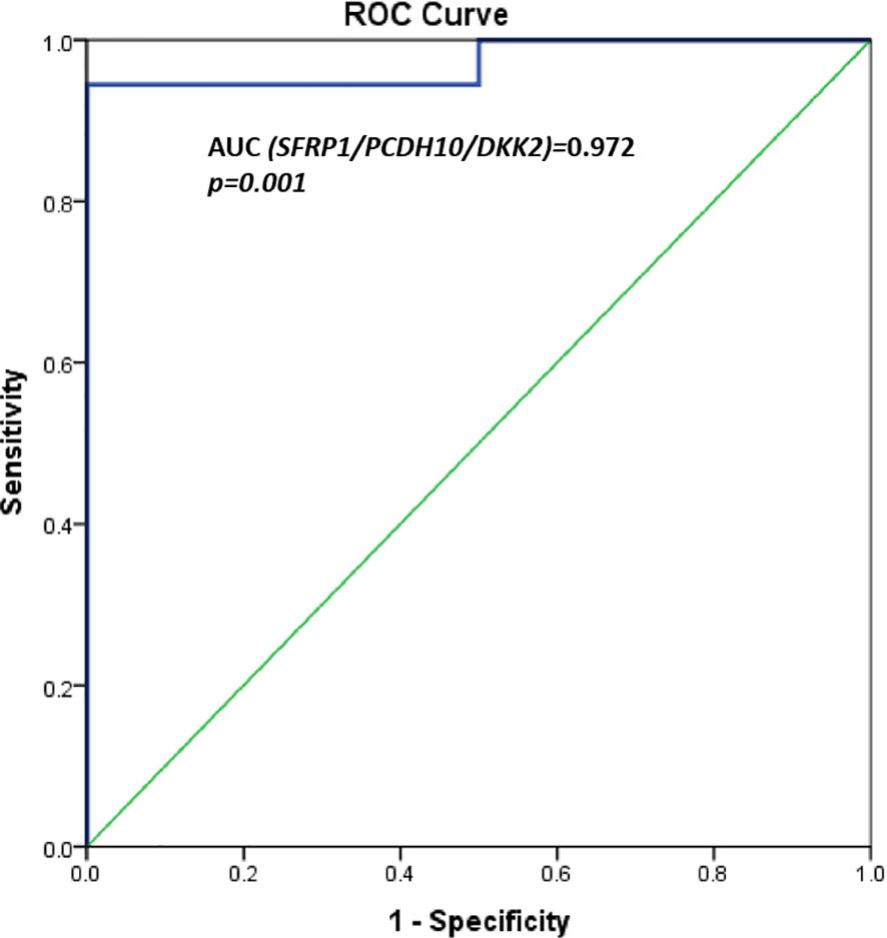
ROC curves for three potential biomarkers combined (SFRP1, PCDH10, and DKK2). AUC = 0.972, p = 0.001.

A significant differential methylation profile of nine genes (*RUNX3*, *SFRP1*, *WIF1*, *PCDH10*, *DKK2*, *DKK3*, *TMEFF2*, *OPCML*, and *SFRP2*) was detected in tumoral tissues when compared to control tissues (**Table 3**). No statistical difference was observed when comparing the methylation levels of NAT to C (*p* > 0.05).To assess the potential diagnostic value in discriminating tumoral from normal tissues from controls, ROC analysis was performed. All the significant genes presented an AUC above 0.80 and a specificity and sensitivity between 77.8 and 100% (**Table 3**). When combining three selected potential biomarkers (*SFRP1*, *PCDH10*, and *DKK2*) the value of the AUC was 0.972 and *p* = 0.001 (**Figure 2**).

**Figure 3:**
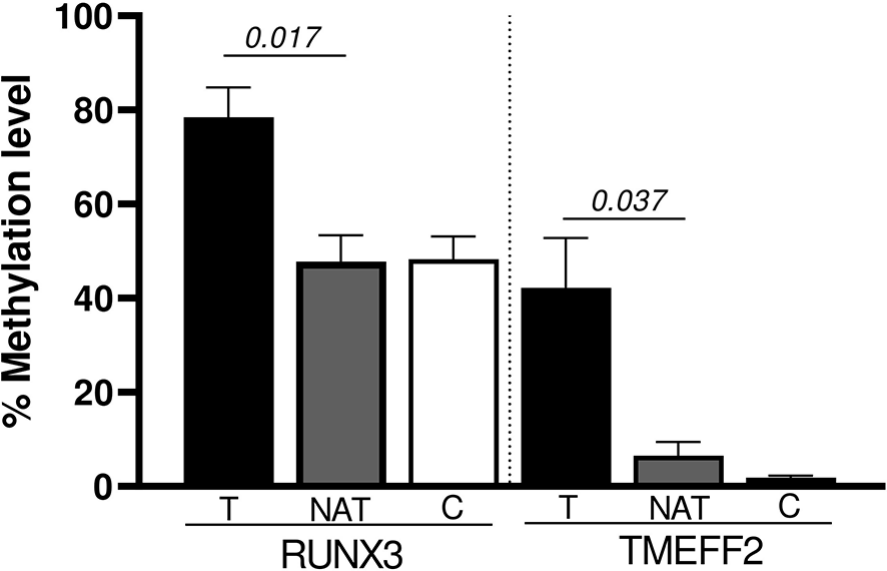
Significant promoter genes hyper-methylated in the paired analysis (Wilcoxon Signed Rank test): T *vs* NAT.

We also performed a paired analysis for the 10 tumoral samples with their matched NATs. The results showed that *RUNX3* and *TMEFF2* promoters were significantly hyper-methylated in tumoral tissue (*p* = 0.017 and *p* = 0.037, respectively) (**Figure 3**). *SFRP1*, *WIF1*, *PCDH10*, *DKK2*, *DKK3*, *OPCML*, and *SFRP2* maintained the same trend of hyper-methylation in T *vs.* C cases, without reaching statistical significance in the comparison with NAT.

**Figure 4:**
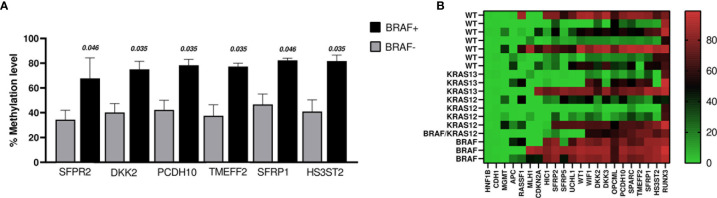
**(A)** The graph shows the genes whose promoters methylation percentage were significantly higher in BRAF+ cases (n = 4) *vs* BRAF− cases (n = 14). The bars indicate the mean ± SEM. **(B)** Heat map of DNA promoter methylation according to mutation status. Methylation levels vary according to the color scale, ranging from light green (low) to red (high).

When comparing methylation profiles according to *BRAF* mutation status, we found that *SFRP2*, *DKK2*, *PCDH10*, *TMEF2*, *SFRP1*, and *HS3ST2* showed methylation levels that were significantly higher in the *BRAF* positive cases (n = 4, including the case with *BRAF*+ and *KRAS12*+) than in *BRAF* negative cases (WT and *KRAS+*; n = 14) (**Figure 4A**). No changes were identified comparing *BRAF*+ *vs* WT (n = 7).

Regarding *KRAS* mutation status, we found that the methylation level of *SFRP2* was lower in *KRAS*12+/13+ (n = 8) *versus KRAS*12−/13− (n = 10) (*p* = 0.027). Even when the case with double mutation (*BRAF*+ and *KRAS*12+) was excluded, the comparison remained significant (*p* = 0.043). Moreover, *OPCML* was less methylated in the comparison of *KRAS*12+ (n = 4) to *KRAS*12− (n = 14), (*p* = 0.035). No significant difference in the percentage levels of methylation was found in the following comparisons: *KRAS*12+ (n = 4) with WT (n = 7), *KRAS*13+ (n = 3) with WT (n = 7), *KRAS*12+/13+ (n = 8) with WT (n = 7). The methylation results of the tumoral samples grouped by mutational status are presented in the heat map of **Figure 4B**.

Furthermore, we quantified the methylation levels of the promoters in peripheral blood mononuclear cells (PBMC) from five controls and five CRC patients whose methylation profiles were performed in the tumoral tissue. None of the five patients presented BRAF/KRAS mutations in their blood. Except for *RUNX3*, which was highly methylated both in patients and controls, the other 21 gene promoters did not show a significant general level of methylation (data not shown).

## Discussion

In this study, we profiled DNA methylation of the promoters of 22 candidate genes in tumoral tissue, normal adjacent tissue from CRC patients, and normal mucosa from controls. In all tumoral samples, we also assessed the mutation status of *KRAS* and *BRAF* to investigate a possible association between hyper-methylation of these candidate genes and *KRAS* and *BRAF* mutations. Our study revealed the hyper-methylation of nine genes out of 22 in tumoral tissue from CRC patients compared to normal mucosa and identified a specific methylation profile for patients with the *BRAF* V600E mutation.

In the case-control comparison, we found hyper-methylation of genes belonging to the Wnt signaling pathway (*SFRP1*, *SFRP2*, *DKK1*, *DKK2*, and *WIF1*), the TGF-Beta;/SMAD pathway (*RUNX3*), two tumor suppressor genes (*PCDH10* and *OPCML*), and a gene that acts both as an oncogene and a tumor suppressor, depending on the cellular context (*TMEFF2*). Many CRCs feature methylation of the extracellular inhibitors of Wnt signaling, such as *SFRP1*, *SFRP2* (13–15), and genes belonging to the dickkopf (Dkk) family. Although genes from the Dkk family largely have an inhibitory effect on this pathway, there is evidence that *DKK2* can also activate Wnt signaling (16). In particular, *DKK2* and *DKK3* were hyper-methylated in tumoral tissue from CRC *versus* control tissues or paired adjacent healthy tissues (13, 17, 18). Furthermore, *DKK3* expression levels were negatively correlated with the rate of promoter methylation (19). Moreover, it has been suggested that *DKK2* and *DKK3* co hyper-methylation might be considered as an independent prognostic predictor (20). Aberrant methylation of *WIF1* was found in CRC (13, 14, 20, 21) with a statistically significant association with increasing tumor stage and tumor differentiation (22).

A high percentage of colorectal cancers contain mutations that disrupt signaling in the pathways of the TGF-β family that regulate the proliferation, differentiation, adhesion, and migration of cells (23). One of the transcriptional effectors involved in TGF-β/SMAD signaling is *RUNX3*. Its promoter has been found to be methylated in approximately 30% of CRCs (15) with high levels both in CRC tissues (17, 24) and in serum, where methylation levels increase with the advancement of pathological stage (25). *PCDH10* belongs to the protocadherin gene family and acts as tumor suppressor gene inhibiting cell proliferation and cell invasion in colorectal cancer development. The rate of *PCDH10* methylation in CRC tissue was significantly higher compared with normal mucosa in different studies (26, 27) as well as in other types of cancer such as lymphomas (28, 29), breast cancer (30) and medulloblastoma (31). Another tumor suppressor gene found hyper-methylated in our study was *OPCML* (Opioid Binding Protein/Cell Adhesion Molecule Like). It is involved in cell growth, invasion, and metastasis. This gene exhibits high promoter methylation and reduced expression levels in different cancers, including ovarian, bladder, nasopharyngeal, and cervical cancers, as well as hepatocellular carcinomas, and colorectal cancer (32, 33). Finally, our comparison between CRC tissue and controls revealed hyper-methylation of *TMEFF2*. In this case, our results are also in line with the literature as it shows aberrant methylation of this gene in CRC (34), suggesting its role as prognostic marker (35).

Mutations in *BRAF* and *KRAS* genes occur in about 10 and 40% of CRC cases respectively (36, 37) and affect different biological pathways. Functionally, these genes are linked to dysregulated DNA methylation (38) and miRNA expression (39). We found a specific methylation profile of tumoral tissues with the *BRAF* V600E mutation that showed increased methylation levels of *SFRP2*, *DKK2*, *PCDH10*, *TMEFF2*, *SFRP1*, and *HS3ST2* compared with tissues without this mutation.

The association of the *BRAF* V600E mutation and *SFRP2* methylation levels has been already shown by Bagci and colleagues (40). In line with the results of this research group, the association between *KRAS* mutations in codon 12 and codon 13 and hyper-methylation of *SFRP2* did not show significant results. Regarding the other identified genes, a specific association between *BRAF* V600E mutation and methylation levels has not, to our knowledge, been reported in the literature.

Epigenetic aberrations are reversible and therefore represent promising targets for novel approaches for cancer therapies (41, 42). The observation that *BRAF*-mutant CRCs displayed six hyper-methylated genes with tumor suppressive functions respect to *BRAF*-negative CRCs may shed light on their possible re-expression following treatment with demethylating agents. In CRC, epigenetic alterations may promote resistance to systemic drugs such as 5-fluorouracil (5-FU), oxaliplatin, and irinotecan (43). For this reason, it has been suggested that combined therapies with epigenetic agents may reverse drug resistance. It has been demonstrated that the use of a demethylating compound, such as 5-azacitidine (5-AC) improves sensitivity and reduces resistance in *BRAF*-mutant CRCs that are usually characterized by very aggressive behavior, poor prognosis, and resistance to therapies with 5-FU or irinotecans (44). Mao et al. demonstrated that treatment with demethylating agents might prime CRC for *BRAF* inhibitor treatment. In a xenograft model of CRC, these authors showed that pretreatment with 5-AC significantly increased the efficacy of subsequent treatments with a *BRAF* inhibitor (45).

Our results indicate a panel of genes that could be considered for the identification of an epigenetic molecular signature of BRAF-mutant CRCs and thus potential molecular targets for selective epigenetic treatments. Several epigenetic drugs are currently under study in clinical trials for the treatment of CRC (46); epigenetic target-based therapy might be a promising approach in the future to improve the curative treatments of CRC.

Clearly, there were some limitations to this study. Firstly, the sample size was relatively small; secondly, mRNA data, which is useful to perform a correlation between methylation and gene expression, was not available.

## Data Availability Statement

The original contributions presented in the study are included in the article/**Supplementary Material**. Further inquiries can be directed to the corresponding author.

## Ethics Statement

The studies involving human participants were reviewed and approved by the Ethics Committee of Victor Babes National Institute of Pathology. The patients/participants provided their written informed consent to participate in this study.

## Author Contributions

MD designed and coordinated the study, performed the laboratory experiments, contributed to data processing and writing. VH and FV were responsible of the collection and diagnosis of the samples included in the study. EM was responsible of data processing, statistical analysis, and wrote the first draft of the manuscript. AS and GP gave their contribution in critical revision, data interpretation, and writing. IAP was involved in scientific writing. All authors discussed the results and commented on the manuscript. All authors contributed to the article and approved the submitted version.

## Funding

This work was funded by the Ministry of Research, Innovation and Digitalization in Romania under grant no. PN 1N/2019_19.29.01.05.

## Conflict of Interest

The authors declare that the research was conducted in the absence of any commercial or financial relationships that could be construed as a potential conflict of interest.
